# Analysis of Uncoupling Protein 2-Deficient Mice upon Anaesthesia and Sedation Revealed a Role for *UCP2* in Locomotion

**DOI:** 10.1371/journal.pone.0041846

**Published:** 2012-08-10

**Authors:** Marie-Clotilde Alves-Guerra, Caroline Aheng, Claire Pecqueur, Sandrine Masscheleyn, Pierre Louis Tharaux, Anne Druilhe, Daniel Ricquier, Etienne Challet, Bruno Miroux

**Affiliations:** 1 INSERM U1016, CNRS UMR8104, Institut Cochin, Paris, France; 2 Université Paris Descartes, Sorbonne Paris Cité, Paris, France; 3 CNRS UMR7099, Laboratoire de Biologie Physico-Chimique des Protéines Membranaires, IBPC, Paris, France; 4 Université Paris Diderot, Sorbonne Paris Cité, Paris, France; 5 INSERM, U970, Paris Centre de recherche Cardiovasculaire, Paris, France; 6 INSERM U845, Centre de recherche “croissance et signalisation”, Paris, France; 7 CNRS UPR3212, Institut de Neurosciences Cellulaires et Intégratives, Département de Neurobiologie des Rythmes, Strasbourg, France; University of South Alabama, United States of America

## Abstract

General anaesthesia is associated with hypothermia, oxidative stress, and immune depression. Uncoupling Protein (UCP2) is a member of the mitochondrial carrier family present in many organs including the spleen, the lung and the brain. A role of UCP2 in the activation of the inflammatory/immune cells, in the secretion of hormones, and in the excitability of neurons by regulating the production of reactive oxygen species has been discussed. Because of the side effects of anaesthesia listed above, we aimed to question the expression and the function of UCP2 during anaesthesia. Induction of anaesthesia with ketamine (20 mg/kg) or isoflurane (3.6%) and induction of sedation with the α2 adrenergic receptor agonist medetomidine (0.2 mg/kg) stimulated infiltration of immune cells in the lung and increased UCP2 protein content in the lung, in both immune and non-immune cells. UCP2 content in the lung inversely correlated with body temperature decrease induced by medetomidine treatment. Challenge of the *Ucp2^−/−^* mice with isoflurane and medetomidine revealed an earlier behavioral recovery phenotype. Transponder analysis of body temperature and activity showed no difference between *Ucp2^−/−^* and control mice in basal conditions. However, upon an acute decrease of body temperature induced by medetomidine, *Ucp2^−/−^* mice exhibited increased locomotion activity. Together, these results show that UCP2 is rapidly mobilized during anaesthesia and sedation in immune cells, and suggest a role of UCP2 in locomotion.

## Introduction

The administration of anesthetics is accompanied with undesired side effects, comprising lung respiratory depression and hypothermia [1]. Although the complete muscle relaxation and therefore the lack of shivering thermogenesis upon anaesthesia explains, to a large extent, the decrease of body temperature, it has been proposed that at least some anesthetics also affect thermoregulatory mechanisms and thermogenic pathways. For instance, the volatile anesthetic isoflurane has been shown to inhibit key metabolic enzymes such as adenylate cyclase and the mitochondrial respiratory complex one, thus impacting the cell energy metabolism. Indeed, metabolic inhibition affects dramatically thermogenic tissues such as brown adipose tissue [2], [3] in which UCP1 mediated uncoupling of respiration to ATP synthesis is used to produce heat [4]. UCP1 is located in the mitochondrial inner membrane and uncouples the ATP synthesis from the respiratory chain by facilitating the re-entry of protons into the mitochondrial matrix. Close relatives of UCP1 (i.e., UCP2 and UCP3) have been isolated in tissues other than BAT [5], [6]. Their mechanisms of action are still under debate and it has been suggested that they are involved in the regulation of mitochondrial dependent fatty acid oxidation and reactive oxygen production [7]. While UCP3 is mainly expressed in muscle, UCP2 is present in many organs and cell types. It is predominantly expressed in the inflammatory/immune system, in white adipose tissue, in the digestive system, in the lung and in some regions of the brain, including the hypothalamus [8], [9]. Bone marrow cell transplantation has revealed that, in the lung, inflammatory/immune cells contribute to 30% of the amount of UCP2 immunodetected in this tissue [10]. Several reports have suggested that UCP2 might indirectly participate in basal or induced thermogenesis. For instance, Walder and colleagues linked the polymorphism of markers in the *Ucp2* locus with resting metabolic rate and energy expenditure during sleep [11]. Gnanalingham et al. have shown that in large mammals, e.g. sheep, UCP2 RNA peaks around the time of birth in the lung [12]. *Ucp2^−/−^* mice are not cold-sensitive, and instead exhibit immune and non-immune phenotypes linked either to oxidative stress or mitochondrial ATP modulation [13]–[16]. Because general anaesthesia is associated with immune depression [17], inflammation [18], hypothermia, mitochondrial perturbations [19] and oxidative stress [20], we aimed to investigate the expression and function of UCP2 upon anaesthesia and sedation.

We report here that anaesthesia and sedation provoke a transient UCP2 upregulation in the lung and a concomitant reversible hypothermia. Locomotion recovery occurred faster in *Ucp2* ^−/−^ mice than in wild type littermates upon arousal from anaesthesia and from myorelaxant induced sedation.

## Materials and Methods

### Animals

Studies on mice were performed in agreement with the institutional CNRS guidelines defined by the European Community guiding principles and by the French decree N°87/848 of October 19, 1987. Authorization to perform animal experiments was given by the French Ministry of Agriculture, Fisheries and Food (A92580 issued February 2 1994, and 92–148 issued May 14, 2002). All protocols were declared and approved by the Necker faculty Animal Care Committee (approval ID 75-738 to BM). Six to eight week-old male mice were used in all experiment. *Ucp2* ^−/−^ mice of C57B6/J genetic background have been previously described [21]. In some experiments, C57B6/J mice were irradiated and then transplanted with bone marrow cells from either *Ucp2* ^−/−^ or *Ucp2* ^+/+^ littermate animals, as previously described [21]. The mice were used two months after transplantation.

### Anaesthesia and sedation

Anaesthesia was achieved using three different methods:

- 3.6% isoflurane (Forene; Abbot, Rungis, France) inhalation for 5 min in an anesthetic chamber (Plexx; AB ELST, The Nederland)
- intramuscular injection (50 µl) of 200 mg/kg ketamine or intravenous injection of 20 mg/kg (Imalgen; Meriel, Lyon, France)
- intramuscular injection (50 µl) of 20 mg/kg ketamine in combination with 0.2 mg/kg of the α2 adrenergic receptor agonist medetomidine (Domitor; Pfizer, Paris, France).

Medetomidine is a myorelaxant that induces sedation and is commonly used to potentiate the effect of anesthetics. In some experiments, when ketamine and medetomidine were co-injected, mice were awoken 15 min after receiving the anesthetic by intramuscular injection (50 µl) of 0.4 mg/kg of the α2 adrenergic receptor antagonist atipamezole (Antisedant; Pfizer, Paris, France). Sedation was obtained by intramuscular injection of 0.2 mg/kg medetomidine. Control mice received 50 µl 0.9% NaCl.

### Physical activity and body temperature measurements

Physical activity and core body temperature were measured in sedated mice implanted with a transponder. Two weeks before the sedation experiment, E-mitter (battery-free) telemetry devices (MiniMitter Co., Sunriver, OR, USA) were implanted intraperitoneally into the mice under gaseous anaesthesia (O_2_/nitrogen protoxide/isoflurane). The mice were recorded using the Vitalview acquisition system from MiniMitter. Alternatively, body temperature was recorded by anal probe measure (Thermocouple probe thermometer CHY 508 BR, Bioseb, Chaville, France). Statistical significance was tested by the two-way ANOVA method with repeated measures, followed by post-hoc Bonferonni t-test (SigmaStat, Jandel). P values<0.05 were considered statistically significant.

### Mitochondria isolation

Mice euthanasia was achieved by cervical disruption. The lung and spleen were collected and immediately immersed in a buffer composed of 10 mM Tris, pH 7.5, 1 mM EDTA, 250 mM sucrose supplemented with protease inhibitors (1 mM benzamidine, 4 µg/ml aprotinin, 1 µg/ml pepstatin, 2 µg/ml leupeptin, 5 µg/ml bestatin, 50 µg/ml sodium-tosyl-phechloromethyl ketone, and 0.1 mM phenylmethylsulphonyl fluoride, all from Sigma, Saint Quentin Fallavier, France. Fresh or frozen tissues were disrupted in a glass homogenizer. Unbroken cells and nuclei were removed by two successive centrifugations of the homogenate at 750 g for 10 min. Mitochondria were collected after centrifugation of the supernatant at 10,000 g for 20 min, and protein content was assayed using a bicinchoninic acid-based kit (Sigma, Saint Quentin Fallavier, France).

### Western blot analysis

Sodium dodecyl sulphate-gel electrophoresis was performed with 30 µg of mitochondrial protein per lane. Blots were incubated with anti-human UCP2 (hUCP2-605, [8]), anti- cytochrome c oxidase subunit I (COX I) or the anti-porin monoclonal antibodies (clones 1D6 and 20B12 respectively; Molecular Probes, Leiden, The Netherlands) and peroxidase activity coupled to the second antibody was revealed using chemiluminescence ECL kit (ECL, GE Healthcare, Little Chalfont, The United Kingdom). COX I and porin are specifically expressed in mitochondria and were used to normalize mitochondria protein content. Direct recording of the chemiluminescence was performed with the charge-coupled device camera of the GeneGnome instrument, and quantification was achieved using GeneSnap software (both from Syngene, Ozyme, Saint Quentin en Yvelines, France). The results were expressed as a ratio of the intensity of the UCP2 band over the intensity of the COX I subunit or porin corresponding band. The switch to porin antibodies was due to the lack of regular supply of COX antibodies. There are no differences between using COX I vs. Porin antibodies as controls; both COX I and porin antibodies gave similar results. **A** Mann-Whitney test was used for statistical analysis. Values are expressed as mean ± sem. P values<0.05 were considered statistically significant. * P<0.05, ** P<0.01 and *** P<0.001.

## Results

### Up-regulation of UCP2 during anaesthesia and sedation in immune and non-immune cells

**Figure 1:**
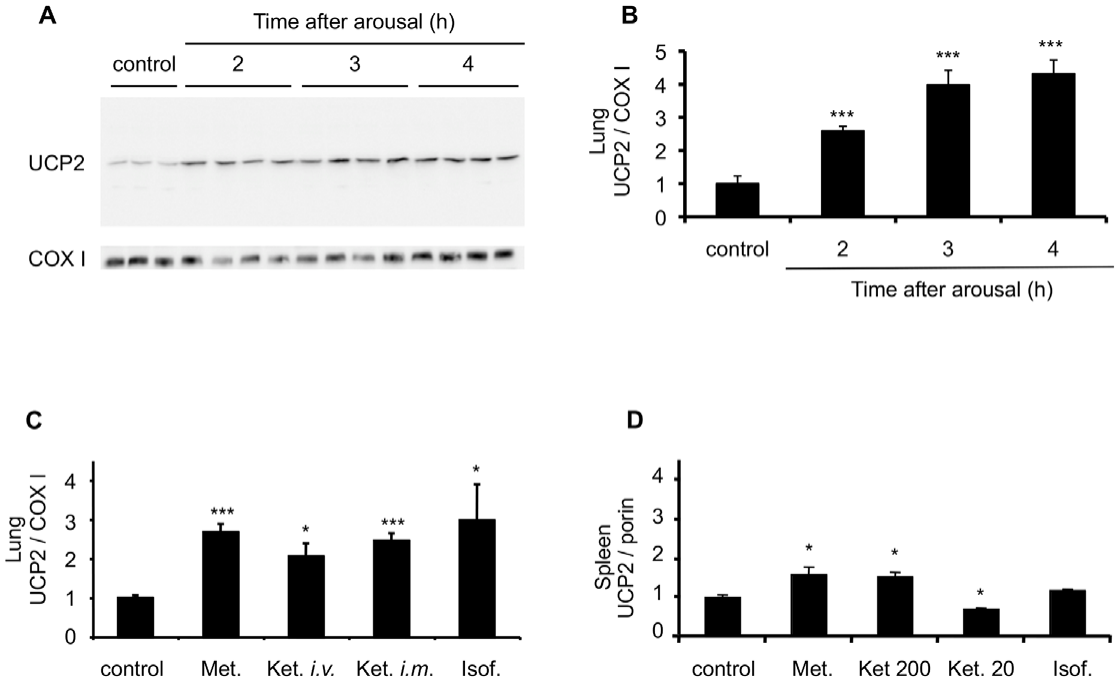
Induction of UCP2 expression in the lung and in the spleen upon analgesia and sedation. **A**, Western blot analysis of UCP2 (upper panel) and COX I (lower panel) expression in the lung of C57B6/J mice. Mice (5 per group) were injected with ketamine (20 mg/kg) in combination with the α2 adrenergic receptor agonist medetomidine (0.2 mg/kg). Fifteen minutes after induction of anaesthesia, the mice were awoken by injection of 0.4 mg/kg of the α2 adrenergic receptor antagonist atipamezole and euthanatized 2, 3 and 4 hours after arousal from anaesthesia. The lungs were collected and mitochondrial preparation and Western blot analysis were performed. **B**, Graphic representation of the UCP2/COX I ratio from the experiment in panel A. **C**, UCP2/COX I ratio in the lung of mice 3 hours after injection of 0.2 mg/kg, medetomidine (Met.), 30 min after intravenous injection of 20 mg/kg of ketamine (Ket. *i.v.*), 3 hours after intramuscular injection of 20 mg/kg ketamine (Ket. *i.m.*), or 3 hours after 5 min inhalation of 3.6% isoflurane (Isof.). COX I subunit was used to normalize the amount of mitochondrial proteins. **D**, UCP2 expression in the spleen of mice 3 hours after injection of 0.2 mg/kg medetomidine (Met.), 200 mg/kg of ketamine (Ket. 200), 20 mg/kg of ketamine (Ket. 20) or 5 min inhalation of 3.6% isoflurane (Isof.). Porin was used to normalize the amount of mitochondrial proteins.

**Figure 2:**
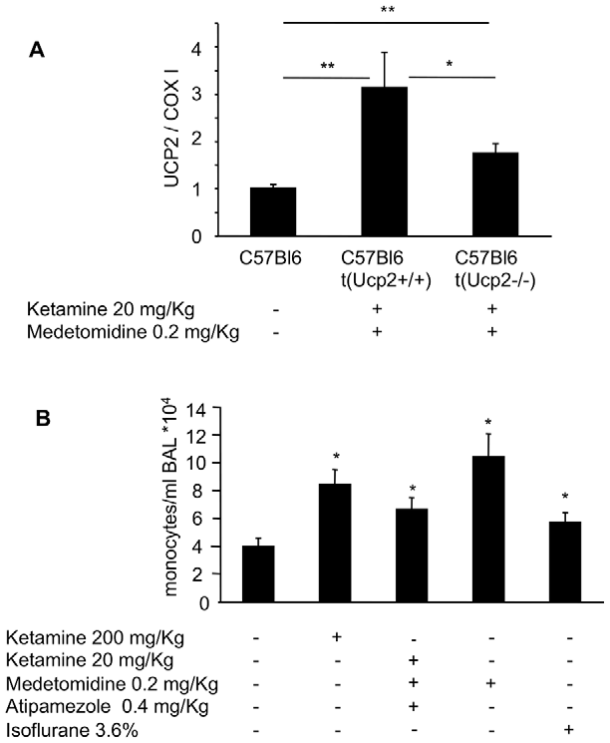
Immune cells contribute to UCP2 expression in lung during analgesia or sedation. **A**, Irradiated C57B6/J mice were transplanted with the bone marrow from *Ucp2^+/+^* (C57B6t(*Ucp2^+/+^*)) or *Ucp2^−/−^* (C57B6t(*Ucp2^−/−^*)) mice as described in (6). Two months after transplantation, the mice were intramuscularly injected with medetomidine (0.2 mg/kg) and ketamine (20 mg/kg). Fifteen minutes after anaesthesia, the mice were awoken by injection of atipamezole and euthanized 3 hours later. UCP2/COX I protein ratio was established by Western blot using the anti UCP2-605 and the anti COX I antibodies. **B**, Three hours after induction of anaesthesia or sedation, the awoken mice were intraperitoneally injected with pentobarbital (50 mg/kg) and bronchoalveolar lavage was immediately performed. Monocytes were identified and counted under a microscope.

In order to test whether UCP2 is upregulated during anaesthesia or sedation, we first tested non-barbituric anesthetics such as ketamine, an antagonist of glutamate receptor. Given the side effects of ketamine on cardio-vascular excitability, ketamine was initially used in combination with the myorelaxant medetomidine (0.2 mg/kg), an α2 adrenergic agonist. Medetomidine potentiated the anesthetic effect of ketamine. Consequently, a sub-anesthetic dose of ketamine (i.e. 20 mg/kg) was sufficient to induce anaesthesia. In those conditions, injection of atipamezole, a competitive α2 antagonist efficiently woke the mice. Following this protocol of anaesthesia/awakening, mice were submitted to anaesthesia for 15 min and a 2 to 4 fold increase of UCP2 protein content in the lung was observed 2, 3, and 4 hours after arousal (Fig. 1A and 1B). In order to dissociate the effect of ketamine and medetomidine, both compounds were injected separately (Fig. 1C). Sedation induced by medetomidine resulted in UCP2 induction. Similarly, UCP2 protein induction in the lung was observed 30 minutes after intravenous injection of ketamine (20 mg/kg) or 3 hours after arousal, from anaesthesia induced by intramuscular injection of ketamine (200 mg/kg). Next, volatile anesthetics were investigated. UCP2 expression increased three fold in lung mitochondria 3 hours after the arousal from isoflurane-induced anaesthesia (5 min-inhalation, Fig. 1C). In contrast, the barbituric anesthetic pentobarbital (50 mg/kg) had no effect on UCP2 expression (data not shown). Given the large *in vivo* distribution of UCP2, tissues other than lung were analyzed. UCP2 protein levels remained unchanged in the digestive system and in the skeletal muscle upon anaesthesia or sedation (data not shown). In the spleen, a more complex pattern of expression was observed (Fig. 1D). Medetomidine increased UCP2 expression of 70% only as compared to the four-fold induction in the lung. Ketamine treatment had dose dependent effects on UCP2 induction. A sub-anesthetic dose of ketamine (20 mg/Kg, intramuscular) decreased UCP2 by 40% in the spleen while a high dose of ketamine (200 mg/Kg, intramuscular) increased UCP2 expression by 60%. Isoflurane had no effect on UCP2 levels in the spleen. Since ketamine and medetomidine modulated UCP2 in the spleen, we tested whether UCP2 induction in the lung was linked to resident or infiltrating blood cells. Anaesthesia was induced on mice previously irradiated and transplanted with bone marrow cells from wild type or *Ucp2* null mice. A three-fold increase of UCP2 content in the lung was observed 3 hours after anaesthesia in animals transplanted with wild type bone marrow cells, while UCP2 expression level increased only by 70% in mice transplanted with the bone marrow from *Ucp2^−/−^* mice (Fig. 2A). The increment of UCP2 expression in animals transplanted with bone marrow cells from UCP2 deficient mice was intermediary between that in non-transplanted mice and in animals transplanted with bone marrow cells from wild type mice, suggesting that UCP2 is increased in structural cells (epithelial, endothelial, smooth muscle cells, and/or fibroblasts) and in resident inflammatory/immune cells (alveolar macrophages). In order to assess whether infiltrating blood cells contributed to UCP2 increase in the lung during anaesthesia or sedation, bronchoalveolar lavages (BAL) were performed. A significant monocytes infiltration was observed upon ketamine, medetomidine or isoflurane treatment (Fig. 2B). No other sign of inflammation was detected. Cytokine levels of MCP1 and IL6 for instance were undetectable in the bronchoalveolar fluids upon medetomidine or ketamine treatment (data not shown).

### Expression of UCP2 correlates with core body temperature during medetomidine induced sedation

**Figure 3:**
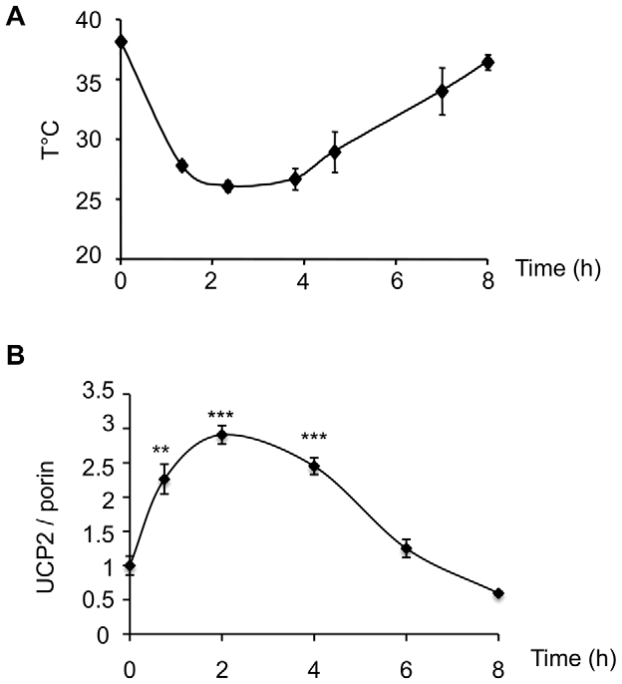
UCP2 induction correlates with sedation-induced hypothermia. Mice were intramuscularly injected with medetomidine (0.2 mg/kg). A, Rectal body temperature was recorded at indicated time after injection. B, Mice (5 on each time point) were euthanized and lung mitochondria were analyzed for their UCP2/porin protein content by Western blot using the anti UCP2-605 and the anti porin antibodies.

Myorelaxants are well known to decrease core body temperature. As shown in Figure 3A, body temperature decreased within the hour following medetomidine injection (0.2 mg/kg). Body temperature returned to its normal level within 8 hours of injection. Given the rapid increase of UCP2 content in the lung upon medetomidine treatment, we performed a complete time course experiment to test if UCP2 expression levels correlate with core body temperature. Interestingly, UCP2 expression levels followed the exact opposite course to core body temperature (Fig. 3B). UCP2 protein content in the lung increased rapidly after medetomidine injection. Maximum expression level of UCP2 remained for 4 hours before it progressively returned to its basal level.

### *Ucp2^−/−^* mice recover faster from sedation or anaesthesia

**Figure 4:**
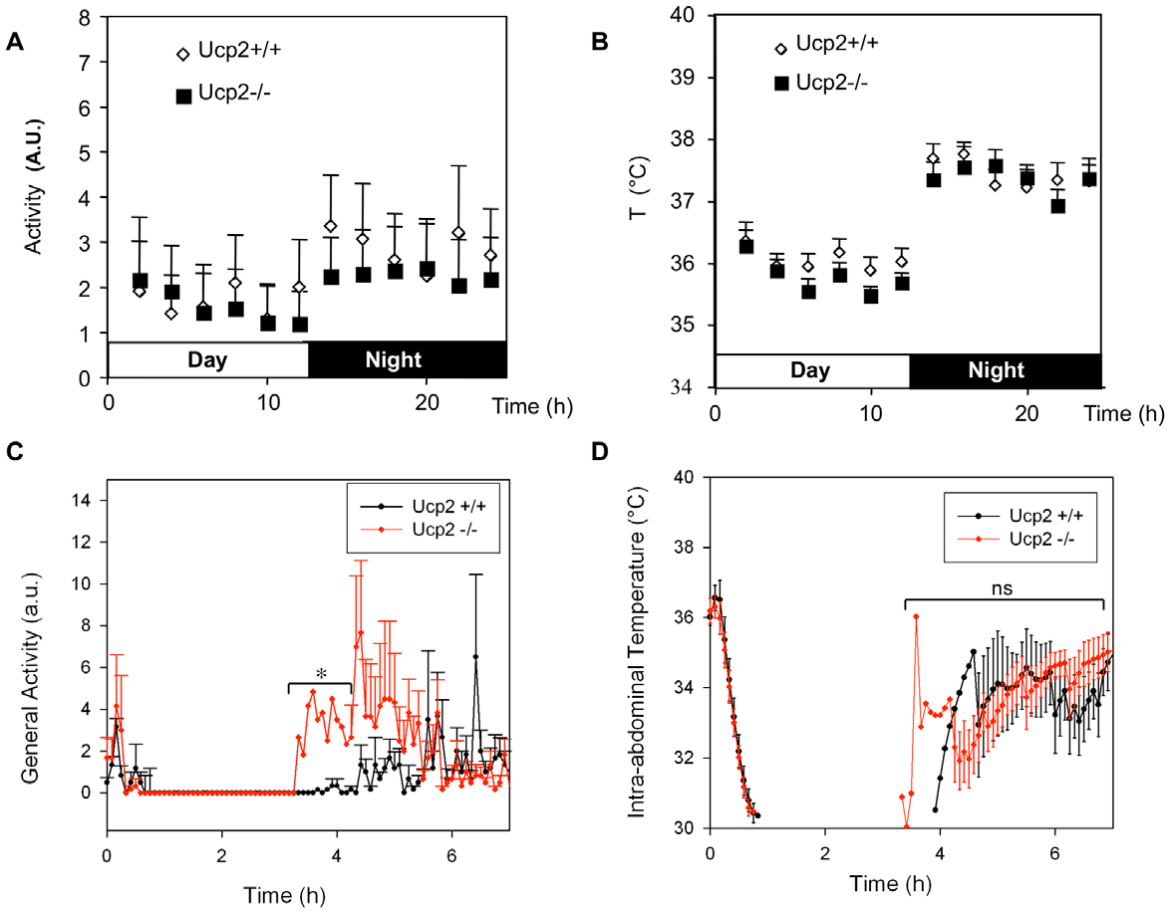
Locomotion and body temperature of *Ucp2^−/−^* mice during medetomidine induced sedation. One week before the start of the experiment, transponders were implanted in mice (five per group). A, Activity and B, body temperature of *Ucp2^+/+^* and *Ucp2^−/−^* 24 hours before medetomidine injection. Data were plotted every 2 hours. Mice were subsequently injected with medetomidine (180 µg/kg) and C, the physical activity and D, the body temperature were recorded every 5 min for 7 hours. Time 0 in panels B and D corresponds to the time of medetomidine injection. Note, using this measurement system, the detection limit of temperature was 30°C. * P<0.05 between genotypes for a given time.

**Figure 5:**
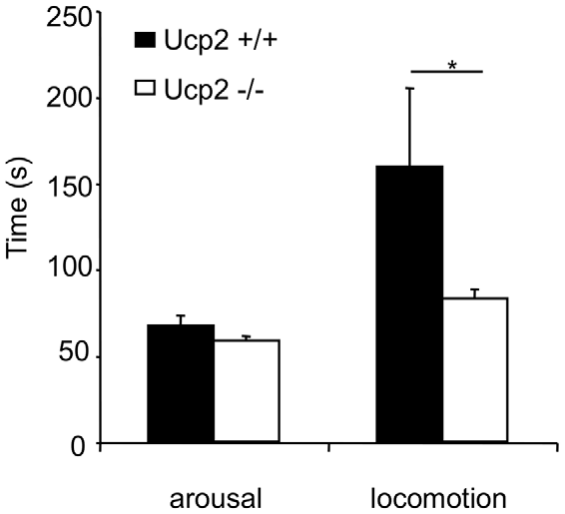
*Ucp2* ^−/−^ mice recover faster than wild type mice upon arousal from isoflurane-induced anaesthesia. Graphic representation of arousal and locomotion times of *Ucp2* ^+/+^ and *Ucp2* ^−/−^ mice after isoflurane anaesthesia. Mice inhaled isoflurane 3.6% for five minutes in an anesthetic chamber. Anaesthesia was interrupted by taking the mice out of the chamber. The mice were laid down on their back and from this time-point, arousal (rotation of the mice onto their feet) and locomotion times were recorded.

In order to assess core body temperature and locomotion activity, transponders were implanted in *Ucp2^−/−^* and *Ucp2^+/+^* mice. Figure 4A–B shows locomotion activity and body temperature of the *Ucp2^−/−^* and *Ucp2^+/+^* in the basal condition, *i.e.* for the whole day before the medetomidine challenge. In contrast to the observation of Andrews and colleagues [22], *Ucp2^−/−^* mice did not exhibit any significant reduction of locomotor activity at night, as indicated by fully overlapping error bars between genotypes for all data points, suggesting that the genetic background (C57B6/J in our study versus C57B6/129SvJ in Andrews's study) strongly influences *Ucp2^−/−^* mice phenotypes. Medetomidine injection provoked a drop in physical activity to zero and a decrease in temperature body to or below 30°C (detection limit of the transponder) within 30 min in both *Ucp2* ^+/+^ and *Ucp2^−/−^* mice (Fig. 4C–D). The activity of *Ucp2* ^−/−^ mice significantly increased 1.5 h before the activity of control mice, and physical activity was increased in *Ucp2* null animals (Fig. 4C). Body temperature recovery was not significantly different in either of the mice genotypes despite a higher locomotion activity in *Ucp2* ^−/−^ mice that was expected to generate heat. To confirm the locomotion phenotype observed in the sedation model, we next studied the recovery phase of *Ucp2^−/−^* mice in the isoflurane induced anesthetic model. Mice were placed in an anaesthesia chamber and submitted to 3.6% isoflurane inhalation. After 5 min of isoflurane treatment, mice were taken out of the anaesthesia chamber and left on their back. Following isoflurane inhalation both *Ucp2* ^+/+^ and *Ucp2* ^−/−^ mice awoke after about 1 min and were immediately rotated to stand on their feet (arousal time). However, once awoken, *Ucp2* ^−/−^ mice needed less than 20 additional seconds to start walking while *Ucp2* ^+/+^ mice remained immobile for 60 s before moving (Fig. 5).

## Discussion

In this study, we show for the first time that UCP2 responds to a physiological change induced by several anesthetics and a myorelaxant. Up-regulation of UCP2 in the lung occurred in less than 1 hour after injection of medetomidine, suggesting a translational control of UCP2 as described in response to LPS, fasting or glutamine treatment [8], [23]. After a plateau of 2 to 3 hours, UCP2 protein returned to its basal level of expression in about 2 hours, which is also in accordance with the short half-life of this protein previously measured [24]. However, inhibition of UCP2 fast turnover cannot be excluded. The general time course of UCP2 induction upon medetomidine treatment is similar to that observed after LPS injection as described in [8], suggesting a common origin. However, although bone marrow transplantation demonstrated that immune cells contribute to the increases of UCP2 in the lung, we could not detect any sign of inflammation in this tissue. In addition, immune cell infiltration in bronchoalveolar fluid was far below that observed during inflammation. It is likely that resident immune cells and especially macrophages increase UCP2 expression. A common side-effect of anesthetics and LPS-induced inflammation in mice is hypothermia [1], [25]. To explore further a putative role of UCP2 in thermoregulation, we performed *in vivo* recording of mouse activity and core body temperature using transponders. We focused on the myorelaxant medetomidine because its action on body temperature is dose-dependent and severe. Transponder analysis after use of medetomidine revealed an earlier locomotion activity upon arousal from medetomidine-induced sedation. We did not observe a significant lack of thermogenic function in *Ucp2^−/−^* mice. However, the core body temperature of *Ucp2^−/−^* mice was not significantly augmented as one would expect following an increase of locomotion activity, and further experiments are required to test if *Ucp2^−/−^* mice encountered thermoregulation problems upon an acute drop of core body temperature. Locomotion activity is a complex behavior that results from the interaction of several neurotransmitter systems, among which the dopamine system appears to be the most important one [26] within the striatum and the accumbens nucleus where UCP2 mRNA is present [9]. In these areas of the brain, medetomidine has been shown to inhibit noradrenaline and dopamine turnover [27]. Since *Ucp2^−/−^* mice exhibit a faster recovery to both medetomidine and isoflurane treatment, we suggest that dopamine metabolism is modified in *Ucp2^−/−^* mice. This hypothesis is consistent with the results of Yamada *et al.* showing that UCP2 over-expression in PC12 cells inhibits dopamine secretion [28], However Andrews and colleagues have proposed that UCP2 does not directly promote dopamine secretion; instead, it controls mitochondrial proliferation, fatty acid oxidation and ROS protection, thus increasing the fitness of dopaminergic neurons [22], [29]. At the molecular level, further investigations are required to assess whether UCP2 acts as an uncoupling protein, thereby preventing ROS formation and limiting ATP supply, or as a metabolic transporter at the interface between several neuropeptides' metabolic pathways.
